# Usage of wild *Oryza* germplasms for breeding in pan-genomics era

**DOI:** 10.1270/jsbbs.24050

**Published:** 2025-02-21

**Authors:** Takanori Yoshikawa, Yutaka Sato

**Affiliations:** 1 National Institute of Genetics, Yata 1111, Mishima, Shizuoka 411-8540, Japan

**Keywords:** wild *Oryza*, genome, National BioResource Project (NBRP), GWAS, neo-domestication

## Abstract

One approach to sustainable agricultural production in a changing global environment is the effective utilization of unutilized germplasms. Among these, crop wild relatives (CWRs) represent valuable germplasms that retain the diversity lost during domestication. The genus *Oryza* has two cultivated species and 22 wild species. One of the cultivated species, *Oryza sativa*, produces the rice that is the staple food for half of the world’s population. We are responsible for the maintenance and distribution of wild *Oryza* genetic resources held by Japan’s National Institute of Genetics (NIG). The NIG has collected the genome sequences of numerous wild *Oryza* accessions, aiming at understanding and promoting the utilization of *Oryza* germplasm for both basic and applied sciences, such as breeding. The genome information of many wild *Oryza* germplasms deciphered by multiple groups is publicly available in databases, allowing for pangenome analysis. This review mainly introduces the wild *Oryza* genetic resources held by the NIG, discusses the genome diversity revealed through genome sequencing, presents new attempts to utilize wild *Oryza* germplasm as novel resources enabled by genome sequencing, and discusses the challenges in further effectively utilizing wild *Oryza* germplasm in breeding.

## Introduction

In recent years, the effects of global climate change on the production of various crops have become apparent (Rezaei *et al.* 2023). This climate trend is expected to continue for the foreseeable future, posing a risk to global food production (Porter *et al.* 2014). Stabilizing crop production is an urgent issue among the United Nations’ Sustainable Development Goals (Campbell *et al.* 2018). Breeding is one of the key measures to address this challenge (Ceccarelli *et al.* 2010). To support this, the isolation of genes related to improved yields and stress resistance and their use in breeding programs are being advanced. Generally, crop stress resistance is governed by multiple quantitative trait loci (QTLs) (Flowers *et al.* 2000, Raj and Nadarajah 2023, Wen *et al.* 2019). Hence, diversity among accessions is a prerequisite for isolating useful genes related to stress resistance and their use. Breeding strategies that enhance resistance traits by accumulating numerous stress resistance genes from diverse cultivars and landraces are commonly used (Joshi and Nayak 2010, Muthu *et al.* 2020).

Over a long period, humanity has substantially improved productivity and established stable food production systems by domesticating wild species (Vaughan *et al.* 2007). Over 10 millennia, wild rice has been domesticated through artificial selection into cultivars highly adapted to cultivation (Kovach *et al.* 2007, Meyer *et al.* 2016, Sweeney and McCouch 2007). However, the domestication process has substantially reduced diversity (Izawa 2022, Zhang *et al.* 2016, and there is no guarantee that enough diversity remains to cope with global climate change.

In contrast, crop wild relatives (CWRs) retain far greater diversity than cultivars and landraces, as they inhabit a wide range of environments. Wild *Oryza* species, including the direct ancestors of cultivated rice, are distributed in low-latitude regions of all inhabited continents (Atwell *et al.* 2014). Their habitats range from brackish water areas exposed to almost seawater-level salinity daily, to regions frequently submerged in deep water, to forest floors (Vaughan *et al.* 2003). Therefore, it is highly likely that they hold stress resistance genes not present in cultivated rice (Gaikwad *et al.* 2021).

Given these characteristics of CWR germplasms, the effective utilization of their diversity is gaining attention. Recently, with the advancement of NGS technology, pangenome analysis, which compares multiple genomes within and between species, has made it possible to quantitatively and qualitatively compare the genetic diversity of CWR genomes with those of cultivated species (Huang *et al.* 2012, Wang *et al.* 2017, Zhao *et al.* 2018). Although there are numerous obstacles to the practical use of CWRs in breeding, their effective use is advancing. Often, the characteristics unique to CWRs causes these obstacles. Understanding adverse traits, such as unsuitability to cultivation, differences in optimal growth conditions among accessions, and reproductive isolation, as well as useful traits, is also essential for their practical use. This review mainly introduces our attempts to link the traits and genomic information of germplasms so as to maximize the utility of wild *Oryza* germplasms in the pangenome era, with a focus on the resources maintained at Japan’s National Institute of Genetics (NIG).

## Wild *Oryza* genetic resources at the National Institute of Genetics

The NIG, founded in 1949 by the then Japanese Ministry of Education (now the Ministry of Education, Culture, Sports, Science and Technology: MEXT) and now a member of the Inter-University Research Institute Corporation under the Research Organization of Information and Systems (ROIS) umbrella, serves as a central hub for life sciences, operating the Genetic Resource Center, the DNA Data Bank of Japan (DDBJ), and a DNA sequencing center. The Genetic Resource Center houses genetic materials such as those of mice, hydra, prokaryotes, zebrafish, *Drosophila*, and wild *Oryza*, and provides them to researchers worldwide.

In the collection, conservation, and distribution of wild *Oryza* germplasms, NIG is supported mainly by the National BioResource Project (NBRP), funded by MEXT (Kurata *et al.* 2010, Sato *et al.* 2021). This service was launched in 2002 by MEXT to promote the use of bioresources in Japan. As of 2024, thirty-four biological resources, including animals, plants, microbes, and cells or DNA materials, are managed across 40 institutions and made available to researchers through the NBRP framework (https://nbrp.jp/en/resource-search-en/). As of June 2024, NIG held 1,725 wild *Oryza* accessions from 21 out of 22 species except for *O. schlechteri* Pilger (KKHH or KKLL) (Table 1). Information about the NIG wild *Oryza* collection is available on Oryzabase (https://shigen.nig.ac.jp/rice/oryzabase/locale/change?lang=en) (Kurata and Yamazaki 2006, Yamazaki *et al.* 2010). Many of these resources were gathered by former NIG professors Dr. Hikoichi Oka and Dr. Hiroko Morishima during 16 exploratory tours worldwide between 1957 and 1993 in 35 countries across Southeast Asia, South Asia, Africa, Latin America, and Oceania (Table 2). Reports from these tours, detailing the collection locations and the habitat descriptions, are also available on Oryzabase (as above).

**Fig. 1.**
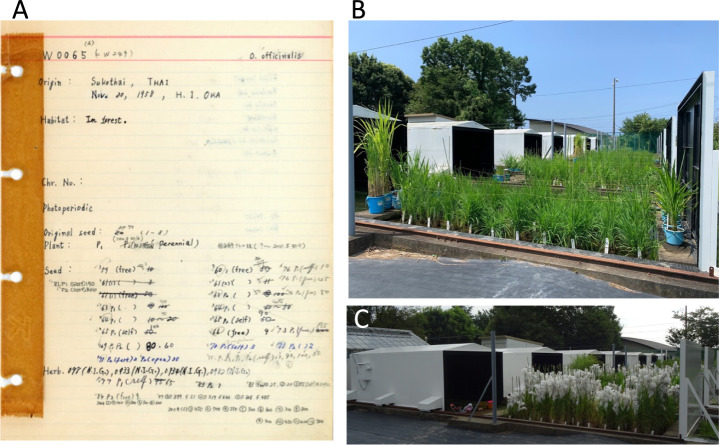
Introduction to the NIG wild *Oryza* accessions. (A) An example of notebook recording history of NIG accessions. (B) Short-day paddy fields in NIG (July). (C) Short-day paddy fields in NIG (August).

Currently, NIG stores original seeds collected since 1957 and the corresponding envelopes, alongside old notebooks containing passport data for each accession (Fig. 1A). Passport data are digitized and are publicly accessible via Oryzabase. Beyond the collections by Drs. Oka and Morishima, additional wild *Oryza* accessions have been introduced through individual researchers or collaborations with research institutions (Table 3). Notably, in the 1980s, NIG and the International Rice Research Institute (IRRI) duplicated many of their wild *Oryza* germplasms. At present, 446 NIG accessions are held by IRRI, and 72 IRRI accessions are held by NIG, each with dual accession IDs in Oryzabase. Users can order these wild *Oryza* germplasms from both IRRI (GRIN-Global: https://gringlobal.irri.org/gringlobal/search) and NIG (Oryzabase). Most of NIG’s publicly available wild *Oryza* resources were acquired before the Convention on Biological Diversity came into force in December 1993.

A notable achievement using wild *Oryza* germplasms in breeding is the introduction of green leafhopper resistance into cultivated rice by IRRI. The resistance gene, derived from *O. officinalis* (IRGC100896/W0065), demonstrates the effectiveness of wild *Oryza* germplasms. Strain W0065 was collected by Dr. Oka in Thailand in 1958 and later transferred from NIG to IRRI (Table 2, Fig. 1A).

## Peculiarities and efforts in *ex situ* conservation of wild *Oryza* resources at the National Institute of Genetics

Most wild *Oryza* species, originating from low latitudes, are short-day plants. NIG, at 35.1°N, has natural long daylength in summer, hindering the flowering of many wild *Oryza* accessions for harvesting mature seeds in the appropriate season. To overcome this, NIG uses a timer-driven large dark box to create short-day conditions in the paddy field during summer (Fig. 1B), facilitating regular seed multiplication. To conduct *ex situ* conservation of wild *Oryza* species in mid-latitude regions such as most of Japan, such efforts and facilities are necessary. To preserve the integrity of genetic diversity in the original seed stock, NIG bags each panicle before flowering to collect self-pollinated seeds and harvests seeds from multiple lines derived from original stocks (Fig. 1C). This means that there are degrees of variation in both phenotype and genotype in accessions due to both heterozygosity and heterogeneity of wild germplasms. However, the increased availability of genome sequence data has increased the demand for inbred lines bred through multiple rounds of self-pollination to reduce the diversity within accessions. For example, Huang *et al.* (2012) sequenced the genomes of a population of 446 *O. rufipogon* accessions (338 from NIG) to argue the origin of domesticated rice. Genomic information and seeds of these accessions are available from Oryzabase. The availability of both genome information and the corresponding biomaterials becomes highly valuable for the efficient use of germplasms in breeding and basic science. This necessitates inbreeding and maintaining correspondence between genomic data and individuals of accessions from which the genomic information is elucidated. Hence, it is important to use, or create, inbred lines of wild *Oryza* species corresponding to reference genomes.

The NIG wild *Oryza* collection contains many perennial strains that do not produce self-pollinated seeds and are maintained via vegetative propagation. Some of these have been preserved by transplanting a couple of times a year for over 60 years, thanks to the dedication of NIG staff. These precious materials are now critical to inferring the heterozygosity of the original populations as well as to reference genome creation.

## Management and utilization of the NIG wild *Oryza* collection

**Fig. 2.**
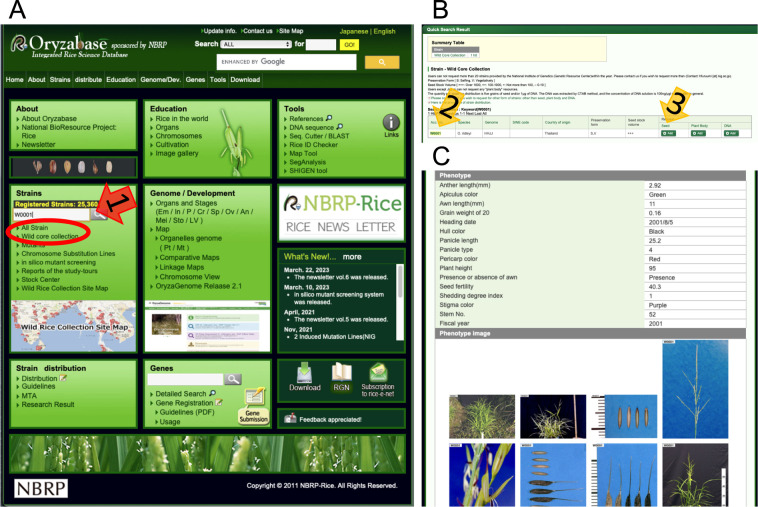
Data access and resource request in Oryzabase. (A) The top screen of Oryzabase (https://shigen.nig.ac.jp/rice/oryzabase/locale/change?lang=en). The NIG wild *Oryza* accessions can be searched from the box pointed with the arrow “1” or by clicking characters in a red circle. (B) In the subsequent screen, users can access to the information of the accession from the link pointed with the arrow “2”, and they can request the seed, plant body, and DNA resources from the link pointed with the arrow “3”. (C) The information page includes biological and phenotypic information, pictures, passport data, and related literatures for the accession.

The NIG wild *Oryza* collection is currently maintained and distributed with the support of NBRP. The NBRP also supports our activities to characterize wild *Oryza*, including genome sequencing, and to operate our databases. Researchers can request over 25,000 biological resources, including wild *Oryza* species, mutants, and experimental strains in Oryzabase (Fig. 2A). Oryzabase provides biological and phenotypic information such as resistance to pests and diseases, passport data, and related literature on accessions (Fig. 2B). Users can order seeds, plants (domestic shipment only), and DNA online (Fig. 2C). Charges include the basic costs for consumables, labor, and shipping, and for quarantine procedures for international shipments. After payment confirmation, the administrative procedure for material transfer begins. Under NBRP policy, users must exchange a Material Transfer Agreement. For shipping overseas, importation and plant quarantine procedures are required. Some countries may additionally require an import permit in advance. In rare cases, resources may not be shipped owing to the inability to meet the specific plant quarantine inspection requirements of a particular country.

From 2018 to 2022, we distributed 3,668 accessions of wild *Oryza* seeds, 143 of plants, and 192 of DNA. Among these, 511 seed accessions (13.9%) and 131 DNA accessions (68.2%) were requested from overseas. Among seed requests, *O. rufipogon* (40.7%) and *O. barthii* (24.9%) are particularly popular. These two species are the direct ancestors of the cultivated species *O. sativa* and *O. glaberrima*, respectively. This suggests that many researchers are interested in the use of CWRs for breeding purposes and understanding domestication. NIG holds both perennial and annual *O. rufipogon* accessions; these characteristics are not discrete but can be intermediate. Thus, NIG does not use *O. nivara* as a species name for annuals closely related to *O. rufipogon* (Liu *et al.* 2015, Zheng and Ge 2010).

In 2022, the top five most popular accessions requested were W1514 (*O. punctata*, 2*x*), W2104 (*O. australiensis*), W1943 (*O. rufipogon*), W0106 (*O. rufipogon*), and W1962 (*O. rufipogon*). The popularity of W1962 is likely due to the availability of chromosome segment substitution lines (CSSLs) that contain chromosome fragments of W1962 within the Taichung 65 (*O. sativa* ssp. *japonica*) genetic background (Yamagata *et al.* 2019). CSSLs for *O. glumaepatula*, *O. meridionalis*, and *O. longistaminata* with the Taichung 65 genetic background are also available (Kurakazu *et al.* 2001, Sobrizal *et al.* 1999, Yoshimura *et al.* 2010). All can be ordered from Oryzabase.

Out of the 1,725 accessions in the NIG wild *Oryza* collection, 282 were selected by passport data as a “core collection” to maximally represent species, geographical origins, and phenotypic and ecological traits (Nonomura *et al.* 2010). The core collection includes 18 species across 9 genome types (AA, BB, CC, BBCC, CCDD, EE, FF, GG, and HHJJ) and is divided into three ranks. Rank 1 (44 accessions) represents the smallest group, selected from all 18 species but containing high diversity, making it suitable for initial investigations of wild *Oryza* species. Rank 2 adds 65 well-studied accessions representing each species. Rank 3 adds another 173 accessions for researchers interested in exploring greater diversity, as these have been used as in the past and have records on several characteristics. Individual accessions can be requested, but these core ranks are highly recommended for researchers who aim at understanding the diversity of wild *Oryza* species with a minimal number of accessions.

## Use of genome sequence information of the NIG wild *Oryza* collection

Currently, genetic information of wild *Oryza* species, including reference genome sequences (ref-seqs), is available in various databases, and Japan’s National Agriculture and Food Research Organization (NARO) publishes SNP information of wild *Oryza* species on TASUKE+ (https://agrigenome.dna.affrc.go.jp/tasuke/ricegenomes/). To offer an open-access platform for genomic data on the highly diverse wild *Oryza* species, we launched OryzaGenome 2.1 (http://viewer.shigen.info/oryzagenome21detail/index.xhtml) (Kajiya-Kanegae *et al.* 2021). This resource includes 217 genome sequences from 19 wild *Oryza* species. It also features an SNP Effect Table derived from 33 deeply sequenced *O. rufipogon* accessions, as well as an SNP Viewer for 446 imputed *O. rufipogon* genomic variants compared with genome assembly IRGSP-1.0. The sequence data for these 446 accessions originate from Huang *et al.* (2012), who proposed a model for the initial domestication of *O. sativa* ssp. *japonica* and *indica* through comprehensive genome-wide analyses of domestication sweeps. Files for tabulated genotype data, VCF files, and PLINK-formatted genotype data for the imputed SNPs of the 446 accessions, available in the Misc Downloads section, can be used for genome-wide association studies (GWAS).

Short-read sequence data obtained over the past few years from more accessions will be made available in OryzaGenome soon. As the short-read data have accumulated, we have noticed that the need to select appropriate ref-seqs for finding both SNPs and structural variations depends on species and sometimes even on subgroups within species. Details on the appropriate selection of ref-seqs are discussed in the next section. So far, 21 *Oryza* ref-seqs cover 12 species in the primary nucleotide sequence repositories, although the quality of the assembly varies from contigs to scaffolds to chromosomes (Table 4). Efforts to produce ref-seqs for wild *Oryza* species have also been carried out under the frame of the International *Oryza* Map Alignment Project (IOMAP), and the number of ref-seq is increasing (https://doi.org/10.1101/2024.05.29.596369), although the quality of the assembly varies from contigs to scaffolds to chromosomes. As OryzaGenome does not yet archive *Oryza* ref-seqs, we are constructing extensive ref-seqs from accessions in the Rank 1 core collection, to be released together with short-read data from as many accessions as possible.

## Genome-wide association study using SNP information of *Oryza rufipogon*

The use of GWAS has rapidly expanded in recent years as a tool to identify genes that cause phenotypic diversity (Wang *et al.* 2016). GWAS analyzes the association between phenotype and genotype by using integrated SNP information of a population, providing high-resolution mapping results without the need for intercross populations. As it is difficult for some wild *Oryza* species to cross with cultivated species owing to reproductive isolation barriers, GWAS can be a useful tool for detecting associated genome regions.

Reductions in the cost of short-read sequencing have made it possible to perform GWAS analysis by using large-scale populations. So far, several GWAS analyses using *O. rufipogon* have been reported. Malik *et al.* (2022) analyzed plant height, culm thickness, panicle length, number of primary branches per panicle, grain length, grain width, and hundred-grain weight by using 346 *O. rufipogon* accessions and detected 19 SNPs located in, or close to, previously reported QTLs or genic regions and 27 novel SNPs significantly associated with traits. Aggarwal *et al.* (2022) analyzed traits related to sheath blight (ShB) by using 405 *O. rufipogon* accessions and identified 22 associated genomic regions, including seven regions overlapping previously reported genes or QTLs for ShB. Their results show the phenotypic diversity of *O. rufipogon* and indicate that it contains useful germplasms for the improvement of cultivated species. Ta *et al.* (2023) analyzed grain size by using 338 *O. rufipogon* accessions, including the Or-I, Or-II, and Or-III subpopulations proposed by Huang *et al.* (2012). They detected 41 QTLs that contribute to the phenotypic diversity of *O. rufipogon* grains, and they found that *qGWT1*, which encodes FT-like 9 (FTL9), has opposite effects on grain size and grain number. They also found a loss-of-function haplotype of *FTL9* prevalent among the Or-III group of *O. rufipogon* and *O. japonica* cultivars, suggesting that large grain size might have been selected in the initial process of rice domestication at the expense of grain number.

**Fig. 3.**
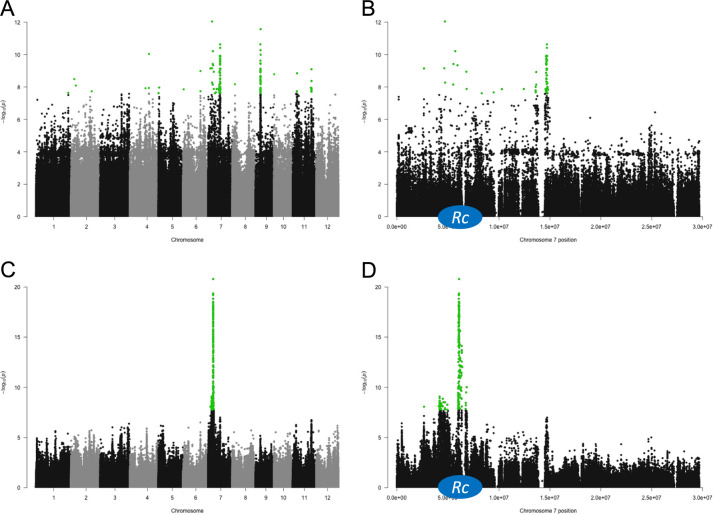
Comparison of the Manhattan plots for the pericarp color in the GWAS analysis using 639 accessions of *Oryza rufipogon*. The 2,047,108 SNP markers used in (A) and (B) were filtered to be higher than 95% of variant call rate, 5% of minor allele frequency, and lower than 5% of heterozygous frequency while 2,853,221 SNP markers used in (C) and (D) were filtered to be higher than 90% of variant call rate, 5% of minor allele frequency, and lower than 0% of heterozygous frequency. The heterozygous genotype in (C) and (D) were replaced with missing value before filtering. The position of *Rc* gene, which is predicted to be the main causative gene for the pigmentation, on Chr. 7 is indicated in (B) and (D), and plots satisfying the threshold of 5% of p-value using Bonferroni correction are highlighted.

GWAS using *O. rufipogon* not only reveals the molecular mechanisms that control the diverse phenotypes, but also provides important findings that unravel the process by which limited haplotypes were selected from diverse germplasms and passed on to cultivated species. At the same time, however, GWAS of *O. rufipogon* poses several difficulties. Relative to *O. sativa*, *O. rufipogon* has large sequence variations, and sequence polymorphisms that do not exist in the *O. sativa* IRGSP-1.0 reference sequence cannot be recovered. Homologous sequences in other regions of the *O. sativa* genome may cause mismapping, resulting in the wrong heterozygous variant call. This mismapping can be reproduced experimentally: when a short-read sequence of Nipponbare (DRR095353) was mapped to a modified IRGSP-1.0 reference sequence in which the coding region of *Tyrosine aminomutase 1* (*TAM1*) was replaced with “missing”, the number of heterozygous variants was increased from 48,207 to 48,272, whereas that of homozygous variants did not change much. Furthermore, 56 of the 65 extra heterozygous variants (86.2%) were concentrated in *Phenylalanine ammonia-lyase 8* (*PAL8*), the homologue closest to *TAM1* (Yan *et al.* 2015), suggesting that the mismapping of *TAM1* reads as *PAL8*. In the case of *O. sativa*, it would be possible to exclude markers containing heterozygous genotypes, but since the degree of genetic fixation of wild *Oryza* species is lower than that of cultivated species, excluding markers containing heterozygous genotypes would greatly reduce the number of markers, reducing the resolution of the results. To tackle this issue, heterozygous genotypes are replaced with missing values and marker bias is eliminated by filtering with minor allele frequency and variant call rate. This modification can significantly improve GWAS results in some cases (Fig. 3). In addition to eliminating uncertain genotype information, it also eliminates the problem of estrangement between the sequenced and trait-investigated generations (the phenotyped offspring may have a different genotype in the heterozygous region of the parent). However, there is a limit to the information that can be obtained by mapping short reads to IRGSP-1.0, so it will be necessary to re-map them to the *O. rufipogon* pangenome.

Major genes detected by GWAS can be introduced into cultivated species by marker selection for breeding purposes. However, where large numbers of small-effect QTLs are detected, it would be not practical to transfer all QTL regions. In such cases, it may be effective to switch from the idea of introducing useful genes from wild species into cultivated species to the idea of domesticating wild species, i.e. neo-domestication. We discuss this idea in the next section.

## Perspectives on the use of wild *Oryza* germplasms in breeding in the post-pangenome era

Wild *Oryza* species are classified into three main complexes: the *O. sativa* complex, the *O. officinalis* complex, and the *O. meyeriana* and *O. ridleyi* complex (Jena 2010, Morishima and Oka 1960). The *O. sativa* complex includes wild species with the AA genome. These are basically cross compatible and allow easy gene transfer from wild species into cultivated species. On the other hand, the *O. officinalis* complex covers the BB, CC, BBCC, CCDD, EE, and FF genomes, and the *O. meyeriana* and *O. ridleyi* complex covers the GG, HHJJ, and HHKK (KKLL) genomes, which have a wide geographical distribution and are cross incompatible with the cultivated species, making gene transfer difficult. Therefore, the way rice germplasms are used will differ between wild species distantly related to *O. sativa* and those closely related to it.

In the case of closely related species, it may be possible to find useful genes and introduce them simply by crossing with cultivated species. So far, yield-related traits, grain quality traits, aluminum tolerance, and grassy stunt virus resistance have been successfully transferred from *O. rufipogon* into *indica* cultivars (Brar and Khush 1997, Nguyen *et al.* 2003, Septiningsih *et al.* 2003a, 2003b, Xiao *et al.* 1996). It has been suggested that genes from *O. rufipogon* could improve the salt tolerance of *O. sativa* (Quan *et al.* 2018). The use of neo-tetraploids also has potential in utilizing the many useful genes of closely related wild species for breeding (Koide *et al.* 2020).

The distantly related wild species have a wealth of valuable traits such as resistance to planthoppers, yellow stem borer, and diseases, adaptation to aerobic soil, and salinity tolerance (Brar and Khush 1997, Ge *et al.* 1999, Khush 1997). The large barriers to using distantly related wild species for breeding need to be overcome. The development of monosomic alien addition lines by using embryo rescue techniques has made it possible to transfer genes of distantly related wild species into cultivated species (Jena 2010, Jena and Khush 1989, 1990). With this method, resistance to brown planthopper and bacterial blight derived from *O. officinalis*, *O. minuta*, and *O. australiensis* has been transferred into cultivated species (Amante-Bordeos *et al.* 1992, Jena and Khush 1990, Multani *et al.* 1994). Nevertheless, the diversity of the genus has not been fully utilized yet.

Neo-domestication is one realistic way to utilize wild *Oryza* species. It is an attempt to domesticate existing wild resources by reproducing the process by which humans first domesticated wild species, through genome editing. Much information has been accumulated in the form of “domestication genes”, which control important traits allowing domestication by artificial selection. One of the achievements of pangenome analysis is the ability to obtain such genetic information comprehensively. So far, attempts at neo-domestication of *O. alta* (CCDD) have been reported (Yu *et al.* 2021), proving that it is possible. Furthermore, as an attempt to explore wild germplasms for another Neo-domestication, the project ‘IOMAP: the Americas’ has initiated to provide a valuable genomic asset describing the genetic diversity, population structure and demographic history, gene flow, and potential hybridization of populations of wild rice species endemic to the Americas (Alsantely *et al.* 2023). A future challenge is how to adapt the transformation technology required for genome editing to a wide range of wild *Oryza* species. We are working on constructing an experimental system for transformation and genome editing by using a wide range of wild *Oryza* germplasms, and we have already transformed and edited the genomes of closely and distantly related wild *Oryza* species, in addition to *O. alta* (Shimizu-Sato *et al.* 2020). On the other hand, there are many wild *Oryza* accessions for which cultivation and gene introduction have not been successful despite extensive efforts to date.

## Challenges for the efficient use of wild *Oryza* germplasms in breeding

The diversity among wild *Oryza* species is extensive. However, the idea that all wild species are superior to cultivated species in traits such as tolerance to all forms of stress is incorrect. It is necessary to properly understand the characteristics of individual wild accessions. This is the most necessary aspect for the active use of wild *Oryza* germplasms for breeding, and it is the area that is largely lacking in the pangenome era. An understanding of both beneficial and disadvantageous traits is required. It is necessary to devise ways to minimize the factors that inhibit human usage while making use of useful parts. Genome information, which has progressed rapidly in recent years, and pangenome analysis by using genome information will be of great help in predicting and controlling the characteristics of wild species. Ultimately, however, it is essential to deeply understand the biological characteristics of individual accessions by using genome information and pangenome analysis in order to utilize wild *Oryza* germplasms for breeding. We hope that many researchers will get their hands on the actual germplasms and observe the traits of interest with their own eyes.

To control the traits of wild *Oryza* species, it is necessary to understand the mechanisms of their expression. The molecular basis of the characteristics of many wild species is difficult to elucidate by using cultivated species or model organisms, so the only option is to use wild species directly as experimental materials. In this case, it is necessary to develop methods for gene isolation from wild species and functional verification through transformation experiments using basic experimental infrastructure tailored to the characteristics of each accession. Access to, and use of, wild germplasms can be a bottleneck. As the NIG wild *Oryza* collection distributes strains that were introduced into Japan before the Convention on Biological Diversity took effect, the hurdles for using these germplasms are low.

Neo-domestication is a reversal of conventional breeding in that it imparts traits of cultivated lines on wild species (rather than introducing wild genes into cultivated lines). In parallel with this approach, it is essential to introduce the superior traits of diverse wild species into cultivated species through conventional breeding by overcoming reproductive isolation barriers. The creation of interspecific hybrids between cultivated species and distantly related wild species will be key to the effective use of wild *Oryza* germplasms in the pangenome era.

## Concluding remarks

We are indebted to Drs. Hikoichi Oka and Hiroko Morishima for collecting the bulk of the NIG wild *Oryza* germplasms. Studying these resources and sequencing their genomes will bring great benefits in rice breeding. Once all of the resources are sequenced, our understanding of the evolution and environmental adaptation mechanisms of *Oryza* species will progress. The use of wild *Oryza* germplasms in breeding has long been a big challenge. We now have the techniques to take advantage of them. Neo-domestication has been shown to be possible. If we can achieve reproductive isolation, we may be able to systematically utilize wild *Oryza* germplasms in breeding. We express our gratitude to Drs. Oka and Morishima for leaving us these valuable resources, and to the many people who have been involved at NIG.

## Author Contribution Statement

T.Y. and Y.S. wrote the manuscript.

## Figures and Tables

**Table 1.** Wild *Oryza* species in NIG collection

Species	2n	Genome type	Origin	Number of accessions
*Oryza sativa* complex				
*O. rufipogon* sensu lato	24	AA	Asia, Oceania	681
*O. barthii* A. Chev.	24	AA	Africa	397
*O. glumaepatula* Steud.	24	AA	Central/South America	99
*O. meridionalis* Ng	24	AA	Oceania	39
*O. longistaminata* Chev. et Roehr	24	AA	Africa	152
*O. officinalis* complex				
*O. punctata* Wall ex Watt, diplo.	24	BB	Africa	10
*O. punctata* Wall ex Watt, tetra	48	BBCC	Africa	10
*O. punctata*		—	Africa	2
*O. minuta* J.S. Presl ex C.B. Presl.	48	BBCC	Asia, Oceania	18
*O. officinalis* Wall ex Watt	24	CC	Asia, Oceania	101
*O. rhizomatis* D.A. Vaughan	24	CC	Sri Lanka	3
*O. eichingeri* A. Peter	24	CC	Africa, Sri Lanka	11
*O. latifolia* Desv.	48	CCDD	Central/South America	31
*O. alta* Swallen	48	CCDD	Central/South America	7
*O. grandiglumis* (Doell) Prod.	48	CCDD	South America	64
*O. australiensis* Domin	24	EE	Australia	37
*O. ridleyi* complex				
*O. ridleyi* Hook. f.	48	HHJJ	Asia, Oceania	6
*O. longiglumis* Jansen	48	HHJJ	New Guinea	13
*O. granulata* complex				
*O. granulata* Nees et Arn. ex Watt	24	GG	Asia, Oceania	7
*O. meyeriana* (Zoll. et Mor. ex Steud.) Baill.	24	GG	Asia, Oceania	19
Others				
*O. brachyantha* Chev. et Roehr.	24	FF	Africa	17
*O. coarctata* Roxb.	48	KKLL	Asia	1
Total				1,725

**Table 2.** Study tours to collect NIG Wild *Oryza* accessions

Tour #	Destination	Year	Collected species
1	India	1957	*O. rufipogon*, *O. officinalis*, *O. granulata* etc.
2	Thailand	1958	*O. rufipogon*, *O. officinalis*, *O. granulata*
3	Philippines and New Guinea	1961	*O. longiglumis*, *O. officinalis*, *O. rufipogon* etc.
4	Latin American countries	1961	*O. glumaepatula*, *O. latifolia*, *O. grandiglumis* etc.
5	Mindanao, Philippines	1963	*O. rufipogon*, *O. punctata*, *O. barthii*
6	Philippines	1963	*O. minuta*, *O. officinalis*, *O. rufipogon*
7	Sierra Leone to Tchad, Africa	1963	*O. longistaminata*, *O. barthii*, *O. brachyantha* etc.
8	Borneo and Java	1963	*O. officinalis*, *O. meyeriana*, *O. rufipogon* etc.
9	East Africa and Madagascar	1964	*O. eichingeri*, *O. longistaminata*, *O. punctata* (2X) etc.
10	Sahara desert, West Africa	1977	*O. longistaminata*, *O. barthii*, *O. punctata* (2X) etc.
11	Tropical Australia	1978	*O. meridionalis*, *O. australiensis*, *O. punctata*
12	Hilly areas of Nepal, India and Thailand	1979	*O. rufipogon*, *O. punctata*, *O. barthii*
13	Thailand	1983	*O. rufipogon*, *O. officinalis*, *O. punctata*
14	Indonesia and Thailand	1985/86	*O. rufipogon*, *O. officinalis*, *O. ridleyi*
15	Bhutan, Bangladesh and Thailand	1989/90	*O. rufipogon*, *O. punctata*, *O. barthii*
16	Amazon basin	1992/93	*O. glumaepatula*, *O. grandiglumis*, *O. latifolia*

**Table 3.** Collection sources of wild *Oryza* in NIG

Collection source	No. of Accessions
Collection tour (1957–1993)	723
Individual researchers	604
IRD, France (former ORSTOM)	132
IRRI, Philippines	72
NARO, Japan (former NIAS)	62
CRRI, Cuttack, Orissa, India	17
Tokyo Univ., Japan	9
USDA, USA	7
Other organization/unknown	99
Total	1,725

**Table 4.** A list of *Oryza* ref-seqs

Species Name.	Genome type	Accession ID	Ref-seq Name	Assembly level	Total length	Assemble ID/ Location
*Oryza rufipogon*	AA	IRGC 106523-1	O. rufipogonIRGC106523RS1	Chromosome	462,580,640	GCA_023541355.1
*Oryza rufipogon*	AA	Taxon A	ASM155180v1	Contig	384,518,444	GCA_001551805.1
*Oryza rufipogon*	AA	W1943	ASM81722v2	Scaffold	339,176,937	GCA_000817225.2
*Oryza nivara*	AA		OnivRS2	Chromosome	395,534,265	GCA_000576065.2
*Oryza meridionalis*	AA	OR44 (W2112)	OmerRS3	Chromosome	393,639,427	GCA_000338895.3
*Oryza barthii*	AA		ObarRS3	Chromosome	347,715,970	GCA_000182155.4
*Oryza barthii*	AA	TB65	LB120v1	Scaffold	295,586,303	GCA_002926215.1
*Oryza barthii*	AA	LB120	TB65v1	Scaffold	294,191,220	GCA_002926235.1
*Oryza barthii*	AA	B88	B88v1	Scaffold	292,234,740	GCA_003020155.1
*Oryza glumipatula*	AA		OgluRS3	Chromosome	388,593,428	GCA_000576495.2
*Oryza longistaminata*	AA	IRGC110404	OL_genome		352,240,197	http://olinfres.nig.ac.jp/
*Oryza longistaminata*	AA	w11	ASM980554v1	Chromosome	371,348,348	GCA_009805545.1
*Oryza punctata*	BB		OpunRS2	Chromosome	422,391,326	GCA_000573905.2
*Oryza officinalis*	CC	W0002	Oryza_officinalis_v1.0	Scaffold	584,134,210	GCA_008326285.1
*Oryza alta*	CCDD	PPR1	PPR1 V1.0	Chromosome	894,549,829	GWHAZTO00000000
*Oryza australiensis*	EE	KR-Keep River_1	MQ-UA-ANU_Oaus-KR_2.0	Contig	858,859,173	GCA_019925245.2
*Oryza brachyantha*	FF		ObraRS2	Chromosome	263,195,077	GCA_000231095.3
*Oryza meyeriana* var. *granulata*	GG	YNKM20150326	ASM399144v1	Contig	776,957,456	GCA_003991445.1
*Oryza meyeriana* var. *granulata*	GG	Menghai	ASM522336v2	Scaffold	736,774,741	GCA_005223365.2
*Oryza coarctata*	KKLL	TKM-2016	OrCoar_V1	Scaffold	569,597,242	GCA_009761635.1
*Oryza coarctata*	KKLL		SCIS-JNU_Pcoar_v1		573,218,882	http://ccbb.jnu.ac.in/ory-coar
